# Epidemiological and clinical characteristics of open globe injuries in Southwest China

**DOI:** 10.3389/fmed.2024.1303683

**Published:** 2024-02-07

**Authors:** Qin Chen, Licong Liang, Yuzhuo Shi, Fang Lu

**Affiliations:** The Department of Ophthalmology, West China Hospital, Sichuan University, Chengdu, China

**Keywords:** open globe injuries, injuries types, intraocular foreign body, Southwest China, ethnic minorities

## Abstract

**Background:**

Open globe injuries (OGIs) are one of the leading causes of monocular vision loss, and the clinical characteristics of OGIs are region specific. The features and patterns of OGIs in Southwest China are poorly known and not well studied. Our study aimed to review the epidemiological and clinical characteristics of patients hospitalized for OGIs in Southwest China.

**Methods:**

A retrospective study of OGI patients admitted to the West China Hospital from January 1st, 2015, to December 31st, 2019, was performed. Demographic characteristics and injury details were recorded. The Birmingham Eye Trauma Terminology system and the ocular trauma score (OTS) were used.

**Results:**

A total of 3,014 patients were included. The male-to-female ratio was 5.2:1, and the mean age was 35.6 ± 19.1 years. 15.2% of patients were from the ethnic groups. The highest-risk occupation was the farmer (30.3%), followed by the worker (28.5%). OGIs occurred more frequently in people with middle (37.0%) and primary school (33.1%) education levels. Types of injuries included 46.8% penetration, 21.2% rupture, 2.9% perforation, and 29.1% intraocular foreign body (IOFB). The injuries types differed between age and occupation groups (*p* < 0.001). IOFBs had a higher risk of causing endophthalmitis, retinal detachment, and traumatic cataracts (*p* < 0.001). The most common injuries resulted from sharp objects (72.7%). The causes of the injuries were significantly associated with age, ethnicity, and occupation (*p* < 0.001). Explosion injuries and attacks by animals were more common among people of Tibetan and Yi ethnicities. Blunt trauma, vehicle crashes, falls and age older than 60 years were risk factors for a lack of light perception and lower OTS scores prognosis.

**Conclusion:**

OGIs in Southwest China mainly affected working-aged males, especially workers or farmers. Severe vision loss and IOFBs are more common findings. OGIs in older patients and ethnic minorities requires additional attention.

## Introduction

Ocular trauma is one of the common causes of vision loss which results in a major economic problem for societies worldwide (1). Open globe injury (OGI), defined as a full-thickness wound of the eye wall, is among the severe types of ocular trauma that can cause irreversible visual impairment and blindness (2). Therefore, OGI imposes a serious financial burden to both individuals and governments due to the additional family care, high medical costs, long-term treatment follow-up and vision rehabilitation, and possible job loss (3). Over the past 15 years, the incidence of OGI has been approximately 2.5–27.7 per 100,000 people worldwide, showing a large variation among countries in which Western countries have a lower incidence than China (4–6). The clinical characteristics of OGI are region specific, for instance, in southeastern Spain, approximately 50% of OGI cases were wire induced (7), while in the midlands of England, more cases were assault-related injuries (8). Since more than 90% of ocular trauma cases are preventable (9), the epidemiological investigation of a given region is very important for the prevention, treatment, and prognosis of OGIs.

Although several previous studies have analyzed the epidemiology of ocular trauma in China (10–14), most data used were collected 10 years ago with few studies specifically investigating the epidemiological characteristics of OGIs in Southwest China. More than 200 million people live in this part of China, and many of them belong to ethnic minorities, including Tibetans. The economic development in this region is not as high as that in the southeastern coastal regions. Therefore, a survey of the epidemiological and clinical characteristics of OGI will provide essential information for OGI management in this region.

West China Hospital of Sichuan University is the largest trauma center in Southwest China, and approximately 7,500 ophthalmic emergencies each year are treated at the hospital. Severely injured ophthalmic events originally occur in Sichuan, Tibet, Qinghai, and Yunnan, and the patients are usually transferred to our hospital. This study aimed to evaluate the common injury causes, associated injuries, and high-risk population groups of OGIs in Southwest China.

## Methods

This was a cross-sectional retrospective study with all data collected from hospitalized patients admitted to the Department of Ophthalmology, West China Hospital from January 1, 2015, to December 31, 2019. All patients had discharged diagnoses of OGI. Our study was approved by the Ethics Committee of West China Hospital and was conducted in adherence with the Declaration of Helsinki and its laws. Because the analyses were performed retrospectively on the deidentified data, informed consent was not required.

The inclusion criteria were an admission diagnosis of OGI according to the International Classification of Diseases, Tenth Revision (ICD-10). Patients who had previously received treatment for OGIs at other hospitals and subsequently presented to West China Hospital due to complications were excluded. All data used in this study were copied from the electronic medical records system, including general information such as age, sex, nationality, occupation, education, delays in presentation, and causes and locations of ocular trauma, and clinical data such as the classification and distribution of ocular trauma, visual acuity (VA) at admission, and accompanying symptoms. In cases of bilateral eye involvement, we collected data selectively from one eye, prioritizing the eye with more comprehensive information, accurate medical records, and a clearer description of the injury status. According to the Birmingham Eye Trauma Terminology system (15), OGI types were subdivided into four categories: penetrating injuries, perforating injuries, intraocular foreign bodies (IOFBs), and ruptures. Based on the location of the globe opening, all OGIs were identified in three zones where injuries occurred. Zone I is involved in an opening of the cornea or corneoscleral limbus, Zone II included injuries taking place at the anterior 5 mm of the sclera, and Zone III covered the full-thickness injuries that extended more than 5 mm posterior to the corneoscleral limbus (16). The ocular trauma score (OTS) was applied to calculate the extent of eye injury and to evaluate the prognosis (17).

### Statistical analysis

All statistical analyses in the present study were performed using SPSS (version 22.0, IBM Corporation, Armonk, NY, United States). Descriptive statistical results were presented as numbers (percentages) for categorical variables and meant for continuous variables. Pearson’s chi-square tests were used to assess correlations between each pair of categorical variables. Cramer’s V and adjusted residuals were used to describe the correlations. All *p* values were nominal and 2-sided, and statistically significant differences were considered at *p* ≤ 0.05.

## Results

### Demographic and baseline characteristics

A total of 3,014 patients were hospitalized with OGIs during the 5-year study period (2015–2019) and included in our analyses. Each year, approximately 7,500 patients with ocular diseases were admitted to the ophthalmic emergency department, and OGIs accounted for 8.04% of ophthalmic emergencies. The baseline data are shown in Table 1.

**Table 1:** Demographics and characteristics of patients for open globe injuries over a 5-year period at the West China hospital of Sichuan university.

Classifications	Cases (*n*)	Percentage
Gender		
Male	2,527	83.8%
Female	487	16.2%
Age groups		
0–6	348	11.5%
7–16	272	9.0%
17–35	722	24.0%
36–60	1,428	27.4%
>60	244	8.1%
Nationality		
Han	2,557	84.8%
Tibetan	276	9.2%
Yi	137	4.5%
Others	44	1.5%
Education level		
Illiteracy	198	6.6%
Preschool period	331	11.0%
Primary school	998	33.1%
Middle school	1,114	37.0%
High school	250	8.3%
University	123	4.1%
Occupation		
Unemployment	299	9.9%
Children	286	9.5%
Student	255	8.5%
Worker	859	28.5%
Farmer	914	30.3%
Non-manual work	401	13.3%
Time of arrival at hospital		
<6 h	523	17.4%
6-12 h	876	29.1%
12-24 h	576	19.1%
1-3d	646	21.4%
3-7d	228	7.6%
>7d	165	5.5%
Total patients	3014	100%

**Figure 1:**
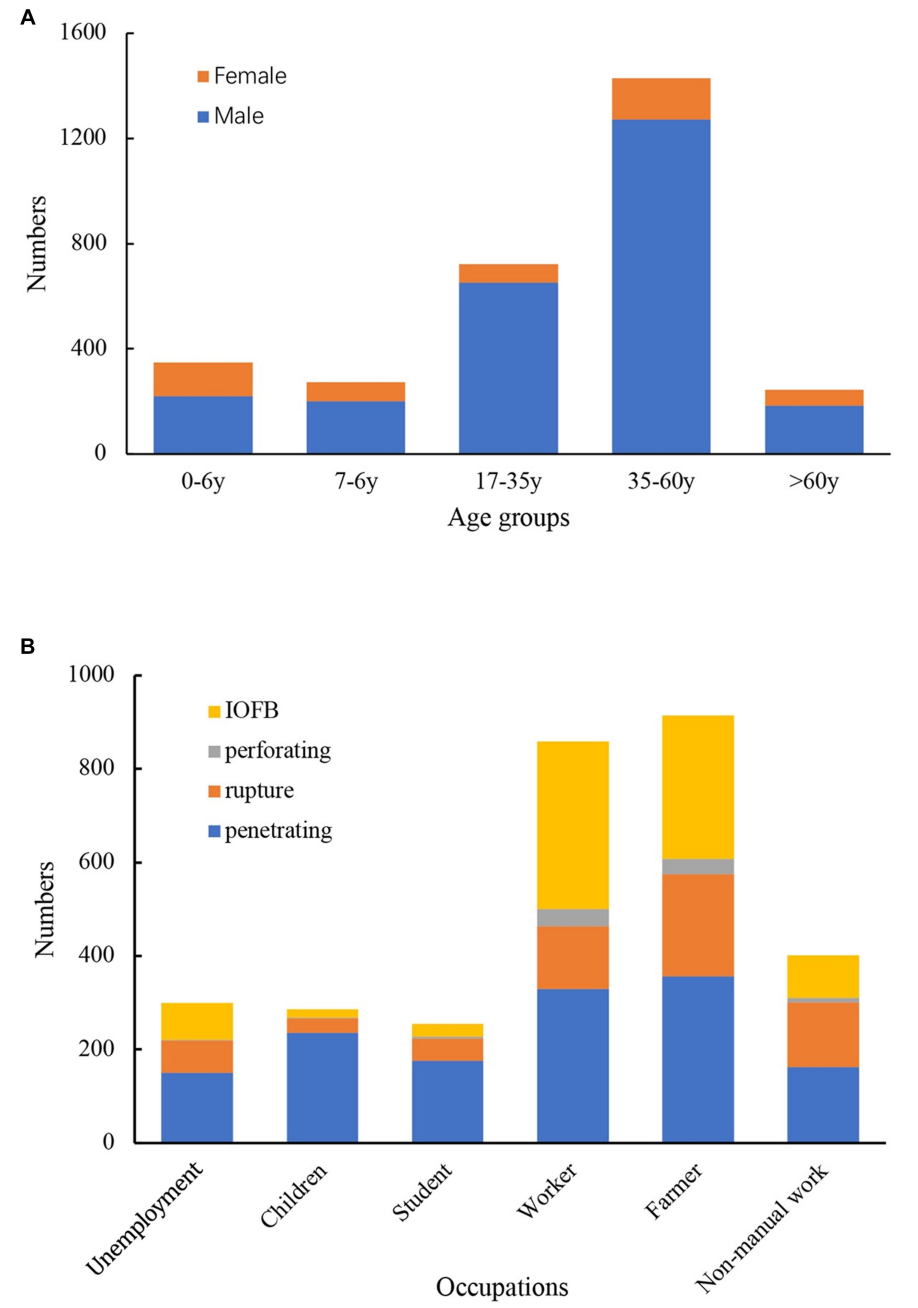
Numbers of OGIs by different classifications. **(A)**, Numbers of OGIs by age and sex. **(B)**, Numbers of OGIs by occupations and types.

Among all patients, 2,527 (83.8%) were male and 487 (16.2%) were female, yielding a male-to-female ratio of 5.2:1. The mean age of the patients was 35.6 ± 19.1 years, ranging from 2 months to 86 years old. There were significant differences in age between sexes, with an average age of 36.6 ± 17.9 years for males and 30.0 ± 23.7 years for females (*F* = 50.7, *p* < 0.001). The general age distributions of the patients were found in 35–60 years (47.4%), 17–35 years (24.0%), and 0–6 years (11.5%). Regarding the highest sex-specific proportion, 90.4% of cases among males occurred in the 17–35 age group, and 36.8% of cases among female occurred in the 0–6 age group (Figure 1A).

The patients were of ethnicities including Han (84.8%), Tibetan (9.2%), Yi (4.5%), and others (1.5%). The age distribution between the combined minorities and those people of Han ethnicity significantly differed (*X*^2^ = 180.2, *p* < 0.001). Among the 3,014 patients, the highest-risk occupation was farmer (30.3%), followed by worker (28.5%). OGIs occurred more frequently in people with middle school (37.0%) and primary school (33.1%) education levels.

**Figure 2:**
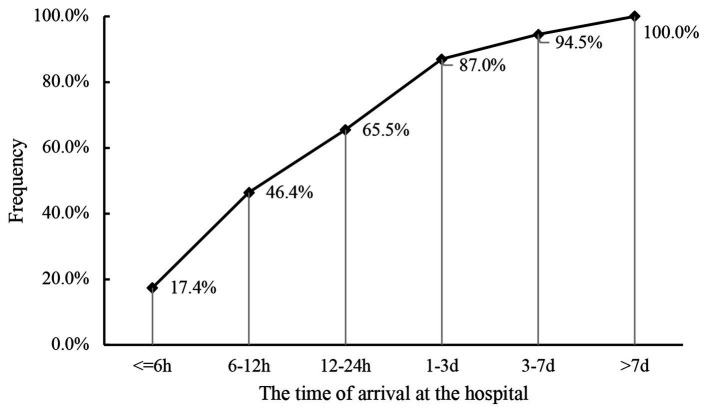
Cumulative proportion of patients at the time of arrival at the hospital.

The delay time from injury happening to hospitalization varied from 1 h to 42 days. Only 17.4% of patients received timely medical treatment in the hospitals within 6 h, 29.1% arrived at the hospitals in 6 to 12 h, and 19.4% arrived in 12 to 24 h, respectively (Figure 2). Unfortunately, only 3.5% of minority patients were provided the medical care within 6 h. The vast majority of minority patients (38.3%) arrived at the hospital in 1 to 3 days. Compared to the low ratio of 10.0% of farmers, 25.8% of workers were able to be hospitalized 6 h after injury.

### Clinical characteristics of OGIs

The various classifications of all OGIs, including the types and locations of the wounds, VA, and concomitant ocular diagnosis, are presented in Table 2. A total of 3,014 eyes were included.

**Table 2:** The injury and ocular characteristics of open globe injuries.

Classifications	Subtype	Cases (*n*)	Percentage (%)
Type	Penetration	1,411	46.8%
	Rupture	639	21.2%
	Perforation	87	2.9%
	Intraocular foreign body	877	29.1%
Zone	I	1757	58.3%
	II	642	21.3%
	III	615	20.4%
Laterality of eyes	OD	1,501	49.80%
	OS	1,493	49.50%
	OU	20	0.70%
Visual acuity	NLP	348	12.3%
	LP/HM	1,464	51.7%
	1/200–19/200	471	16.6%
	20/200–20/50	355	12.5%
	>20/50	191	6.8%
	Missing data	185	6.1%
Complications	Endophthalmitis	607	20.1%
	Retinal detachment	319	10.6%
	Choroidal hemorrhage	97	3.2%
	Hyphtma	988	32.8%
	Vitreous hemorrhage	1,063	35.3%
	Traumatic cataract	1788	59.3%
	Lens dislocation	306	10.2%
	Facial or orbital fracture	175	5.8%
	Eyelid and lacrimal duct laceration	439	14.6%
OTS scores	I(0–44)	586	19.4%
	II(45–65)	961	31.9%
	III(66–80)	881	29.2%
	IV(81–91)	228	7.6%
	V(92–100)	137	4.5%
	missing data	221	7.3%

The types of injuries were different between age groups, and this difference was statistically significant (*X*^2^ = 405.1, *p* < 0.001). In patients under 16 years old, penetrating injuries accounted for 78.1%, which was much higher than the proportion in people (38.7%) over 16 years old. IOFBs occurred most frequently in the ages of 17–35 (36.7%) and 35–60 (35.9%) age groups. The rate of rupture was the highest among patients over 60 years of age (44.7%). Between job types, the type of injury significantly differed (*X*^2^ = 361.6, *p* < 0.001). Workers and farmers experienced the most IOFBs (41.8 and 33.5%, respectively; Figure 1B).

No light perception (NLP) was the most severe visual impairment, and 23.1% of patients older than 60 years suffered NLP after OGI. Different injury types were correspondingly related to typical eye complications. The results of *post hoc* testing among the groups are shown in Table 3. The OTS scores, an index of effective reorganization and evaluation for the prognosis of OGI, were correlated with age, hospital arrival time, and education level. Among those patients older than 60 years, 33.1% had an OTS score of 0–44, while only 0.4% had an OTS score of 91–100. Among patients with an OTS score of 0–44, the arrival time at the hospital was approximately 1–3 days (26.6%), and this rate decreased when arrival time to the hospital was less than 6 h (adjusted residuals was −4.2). The proportion of patients with an OTS score of 91–100 was relatively high (10.1%) for patients who had been hospitalized for more than 7 days. Regarding OTS scores, 30.8% of illiterate patients had a low count of 0–44, while 35.4% of illiteracy had a medium count of 45–65.

**Table 3:** Injury type related to concomitant ocular diagnosis.

	Penetrating		Rupture		Perforating		IOFB		*χ* ^2^	*P*
Endophthalmitis	283	(−0.1)	24	(−11.6)	16	(−0.4)	284	(10.7)	188.558	<0.001
Retinal detachment	70	(−9.4)	63	(−0.7)	21	(4.2)	165	(9.4)	127.154	<0.001
Choroidal hemorrhage	29	(−3.4)	30	(2.4)	9	(3.8)	29	(0.2)	24.808	<0.001
Hyphtma	338	(−9.7)	468	(24.5)	38	(2.2)	144	(−12.3)	635.808	<0.001
Vitreous hemorrhage	258	(−18.3)	432	(19.3)	70	(9.0)	303	(−0.5)	548.981	<0.001
Traumatic cataract	900	(4.7)	204	(−15.9)	62	(2.3)	622	(8.3)	264.469	<0.001
Lens dislocation	92	(−6.2)	168	(15.2)	6	(−1.0)	40	(−6.5)	233.930	<0.001
Facial or orbital fracture	24	(−9.0)	131	(17.9)	4	(−0.5)	16	(−6.0)	321.426	<0.001
Eyelid and lacrimal duct laceration	130	(−7.8)	239	(18.4)	8	(−1.4)	62	(−7.5)	341.898	<0.001

Adjusted residuals appear in parentheses to the right of observed numbers.

### Causes of injury

Overall, the most common injury resulted from sharp objects (72.7%), followed by blunt injury (13.3%). The causes of injuries were significantly associated with age, ethnicity, and occupation (*p* < 0.001). The rates of sharp injuries were the highest among workers and farmers, at 80.8 and 74.2%, respectively. Among sharp injuries, most were caused by metal materials, with a 7:3 ratio of metal objects to nonmetal objects. Sharp injuries were moderately correlated with the age (Cramer’s V = 0.324, *p* < 0.001) and weakly correlated with occupation (Cramer’s V = 0.293, *p* < 0.001). The results of *post hoc* testing among the groups in this study are shown in Table 4.

**Table 4:** Crosstabulations of sharp instrument type and age, occupation groups.

	Age groups (*χ*^2^ = 907.786, *P* < 0.001)					Occupation groups (*χ*^2^ = 930.400, *P* < 0.001)					
Type of Sharp instrument	0-6 y	7-6 y	17-35 y	35-60 y	>60 y	Unemploy-ed	Child	Student	Worker	Farmer	Others
Nail	5(−6.8)	7(−5.0)	106(2.1)	241(7)	8(−3.5)	20(−3)	3(−6.3)	5(−4.9)	193(9.3)	116(0.4)	30(−1.6)
Scissors, knife and needle	94(17.4)	31(4.3)	33(−2.1)	19(−10.9)	5(−2)	27(2.5)	84(17.5)	24(3)	12(−7.7)	16(−6.7)	19(0)
Other metal instrument	46(−9.2)	27(−8.5)	272(3.5)	561(7.6)	60(0)	66(−4)	36(−8.3)	31(−6.9)	387(7.3)	339(3.9)	107(0.9)
Stone, wood and bamboo	48(1.1)	43(2.7)	54(−4.5)	158(−1.8)	52(7.2)	50(3.1)	36(0.4)	35(1.8)	57(−7)	154(5.6)	23(−2.7)
Ceramic, glass and plastic	38(3.2)	30(3.4)	57(1.2)	72(−4.1)	7(−1.7)	29(2.3)	31(2.9)	28(3.5)	41(−3.8)	35(−4.4)	40(4.5)
Pen and pencil	21(4.9)	43(16.5)	2(−4.2)	2(−7.7)	0(−2.2)	13(2.7)	13(2.7)	38(15.3)	1(−5.5)	0(−5.6)	3(−1.7)
Limbs or animals	4(1.1)	2(0.2)	6(0.6)	6(−1.7)	2(0.7)	4(1.6)	4(1.6)	2(0.4)	0(−3.1)	6(−0.1)	4(1.4)

Adjusted residuals appear in parentheses below observed numbers.

**Figure 3:**
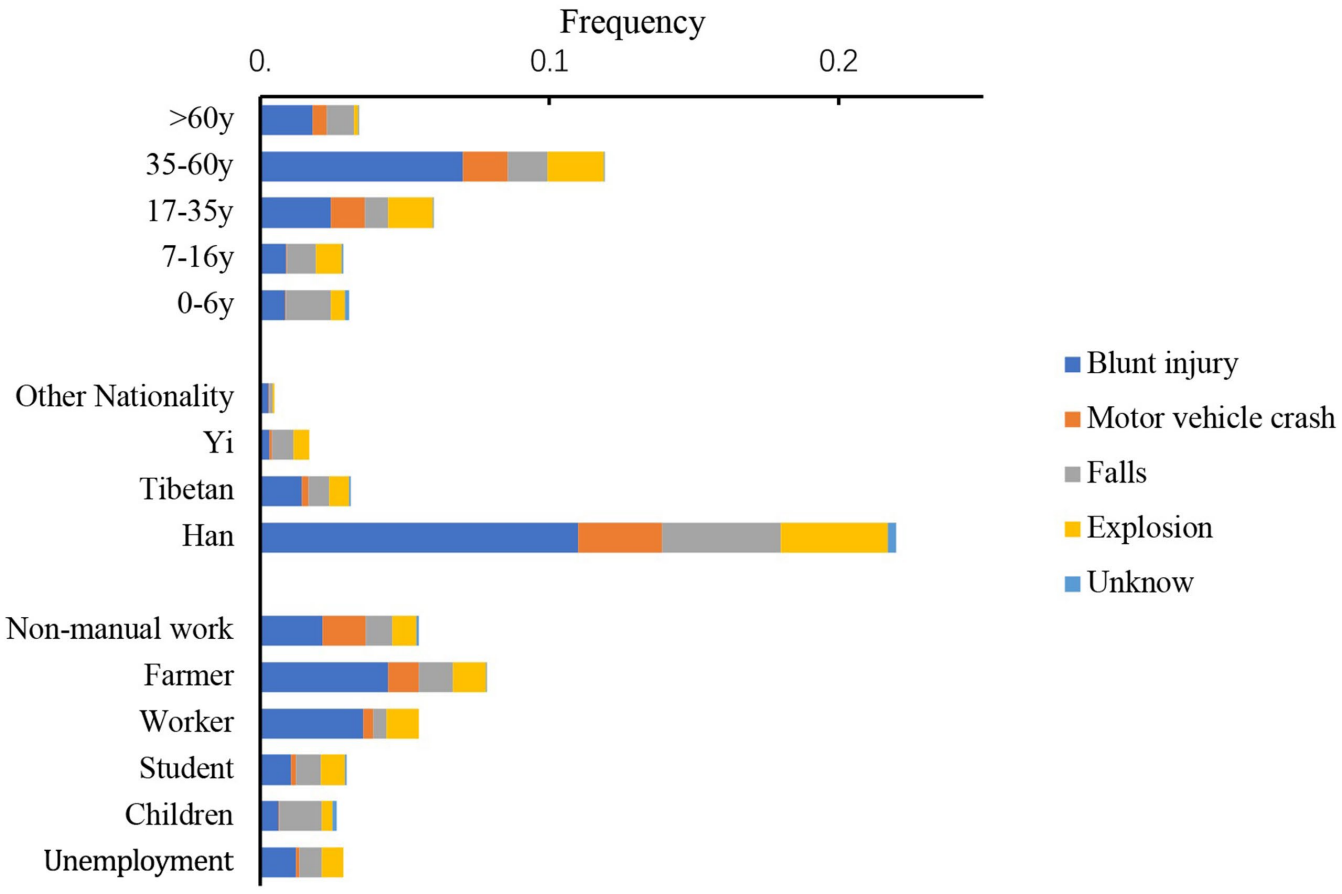
Incident frequency of OGI is categorized by age, nationalities, occupation, and causes of injury (except sharp injury).

Blunt trauma was most likely to occur in people over 60 years old, accounting for 22.5% of cases in this category. The highest rate of OGIs from traffic accidents was found among nonmanual workers. A few blunt force injuries (10.9%) were due to being beaten by a fist. For the 0–6 and > 60 age groups, the proportions of injuries caused by falls were 13.5 and 11.9%, respectively, while for the 17–60 age group, this proportion decreased to approximately 3%. Explosion injuries occupied 7.6 and 11.7% of all cases among Tibetans and Yi ethnicities, respectively, and only 4.4% of cases among Han ethnicities (Figure 3). Explosion injuries caused by fireworks accounted for 38.4%. Two patients suffered eye injuries resulting from gunshots, and the other blast injuries were due to items such as detonators, lighters, gas tanks, light bulbs, and pressure gages. Among all injuries, 3.1% of patients explicitly stated that the injury was related to violent attacks. Twenty-eight patients were attacked by animals, and the proportion of OGIs from animal attacks in ethnic minorities was significantly higher than that in Han people (χ2 = 16.852, *p* < 0.001).

Differences in causes of injury could lead to different types of damage, initial VA, and prognoses. Seventy percent of binocular OGIs were attributed to blast injuries. Sharp trauma was correlated with a better initial VA of 20/200–20/50 and > 20/50 (adjusted residuals were 7.1 and 4.2, respectively). Blunt trauma (33.7%), vehicle crashes (34.7%), and falls (31.3%) were relative risk factors for NLP (adjusted residuals were 13.7, 6.9, and 7.1, respectively), as well as a lower OTS score prognosis (adjusted residuals were 16.2, 6.2, and 5.4, respectively).

## Discussion

This was a retrospective analysis of OGIs in the last 5 years before the coronavirus disease 2019 epidemic era. The patients included in this study lived in Sichuan Province, the Tibetan Autonomous Region, and Yunnan Province in Southeastern China. The results reflect the characteristics of OGIs under the nonepidemic condition in this region.

Consistent with previous studies, our study confirmed that OGIs occurred mainly in the 17 to 60 working-aged group, and the majority of patients were males (18, 19), probably due to more males being engaged in manual labor-related jobs and paying less attention than females to trauma protection. Compared with other regions in China (20), the injured population in the southwestern region had lower educational levels, with 70.1% of patients just having a junior and primary school education level. This may result from the lower economic and educational developments in the southwestern region. Our study demonstrated that the occupations that suffered the most OGIs were farmers and workers because Southwest China is an important agricultural and industrial region. Many studies have shown that ocular trauma could be effectively reduced through enhancing education and improving protective measures (21, 22). Therefore, eye protection training and equipment configuration for workers and farmers will help decrease the incidence rate of OGI.

Timely treatment is very important to minimize eye trauma. Previous studies, as well as our study, have shown that the earlier the medical care is carried out, the better the visual prognosis is (23, 24). However, only 17.4% of patients were able to arrive at the hospital within 6 h for medical intervention in our study. Among these patients, the ratio of workers was significantly higher than that of farmers and other occupations. Among those patients who arrived at the hospital within 1–3 days, farmers and ethnic minorities accounted for a relatively larger proportion because these people lived in areas far from the trauma center or in remote mountainous areas where transportation was inconvenient. These patients have low education levels and poor personal health awareness. Nevertheless, 26.6% of the patients who arrived at the hospital within 1–3 days had OTS scores of only 0–44, which indicated a poor visual prognosis. Therefore, for farmers and ethnic minorities, improving the transportation system, promoting the local medical level, and strengthening education on the dangerous awareness of eye trauma is essential for better visual prognoses.

Compared with other domestic and international data, our study showed the highest incidence rate of IOFBs, up to 29.1% (10, 11, 19, 25). The situation might be attributed to uneven medical resource distributions in Southwest China (26), resulting in a few local ocular trauma centers that can perform vitrectomy or other surgical treatments for IOFBs. Therefore, most patients in this area were forced to concentrate in the West China Hospital. The occurrence of IOFBs is often associated with work-related injuries (1, 12). Most people who are injured in the work environment not only disregard the use of protective eye equipment but also lack safety consciousness and risk awareness. Almost all patients we interviewed did not use protective measures such as goggles while knocking or working with tools. This finding is consistent with those of many studies conducted in areas with poor development levels (27, 28). A study in Taiwan indicated that eye protection devices could reduce the risk of work-related eye injury by up to 60% (29). IOFBs were closely related to severe blinding ocular complications: endophthalmitis, retinal detachment, and traumatic cataracts. Therefore, corresponding laws and regulations should be formulated to force employers to equip each high-risk worker with protective measures.

In the elderly population older than 60, the rate of rupture injuries was as high as 44.7% (the rate of rupture injuries in the general population was 21.2%). Because of poor visual contrast sensitivity, poor depth perception, impaired cognitive function, and high incidence of bone and cardiovascular and cerebrovascular diseases (30), elderly people have a higher risk of severe falls and injuries while moving heavy objects. Moreover, our study showed that 23.1% of the elderly people had NLP after injury, and 33.1% had an OTS score of only 0–44 points. Visual outcomes from the ocular trauma are worse in older adults (31). Rupture injuries are more often accompanied by orbital facial fractures, eyelid or lacrimal ductule laceration, etc., which require greater expenditures and multiple treatments. With the aging of society as a whole, more attention should be given to ocular trauma in elderly individuals, and targeted protection will help avoid serious visual impairments and economic burdens.

Different causes of injury also lead to different visual outcomes, such as blunt trauma, vehicle accident injuries, and falls, which are associated with severe visual impairment. Understanding the cause of injury is crucial to preventing and controlling OGIs. Sharp object injuries were the main cause of OGIs in Southwest China, and the proportion of this cause was higher than that in previous studies (32, 33). Among those sharp injury cases, OGIs in children were usually related to the use of scissors, knives, and other tools, while OGIs in the school-age population were mainly due to pencils. Nails were a common cause of injuries for workers, especially when nail guns were involved. Notably, nail guns often hit the posterior segment of the eyeball, which can irreparably damage vision (34). Except for sharp metal objects, ocular trauma in farmers was associated with sharp objects in nature, such as stones, twigs, and bamboo. Blast injuries are unique cases in China because there is a tradition of setting off fireworks and firecrackers on holidays. However, explosive injuries accounted for only 5% of all injury causes in our study, far lower than the 11.1–24.5% in previous studies. Among explosive injury causes, fireworks and firecrackers accounted for only 38.4% of blast trauma, which was the lowest proportion in the Chinese population (10, 12). It is likely that the low rate of injury directly results from the gradual implementation of fireworks and firecracker safety management regulations in China in 2015. Laws and regulations have played an effective role in reducing special types of eye injuries.

According to the seventh national census of China in 2020, there are 125 million people who belong to an ethnic minority, accounting for 8.89% of the population. Sichuan Province and Southwest China are the main areas where Tibetan and Yi people live. Therefore, the proportion of ethnic minorities with OGI cases was relatively high (15.5%). Compared with Han patients, Tibetan and Yi patients were injured at younger ages, took longer to reach the hospital, and had more falls and explosion damage. Since the lifestyle of ethnic minorities is dominated by agriculture and animal husbandry, there were more animal injuries compared with the Han population. This is the first study to include OGI data on ethnic minority populations in China. The results suggest that for ethnic minorities, multiple steps should be considered, such as enhancing local medical levels, advertising the hazards of ocular trauma and medical care knowledge in daily life, and especially educating children and adolescents on how to prevent OGIs.

This study has several potential limitations that can be topics for future studies. First, all data included were collected from the West China Hospital, which cannot fully represent the whole region. Second, since the electronic medical records system had changed, we were unable to conduct the long-term follow-up of visual outcomes, and instead, the OTS scores were used to estimate prognoses. In practice, many studies have shown the validity of the OTS score for visual prognostic assessment (35, 36). Meanwhile, because most of the patients could not provide prognosis information, we were unable to analyze the straightforward link between OGIs and socioeconomic parameters. Last, the relevant descriptions, parameters, and signs recorded in the old documents may not be accurate. For example, some children were unable to comply with the instructions to achieve an accurate measure of initial VA. In addition, the judgment of relative afferent pupillary defect was inaccurately or unclearly recorded, resulting in a lack of the corresponding OTS score. However, even if relative afferent pupillary defect could not be precisely evaluated, the OTS was still a reliable prognostic tool for ocular trauma (37).

In summary, our study analyzed the epidemiological trends, ocular characteristics, and causes of injury among 3,014 patients with OGIs in Southwest China. Ocular trauma in this region mainly affected working-aged males, especially workers or farmers. The proportion of IOFBs was higher than in the eastern parts of China, and severe vision loss was more frequently found. The OGI characteristics of elderly patients and ethnic minorities showed unique patterns. According to our results, improving local medical care, increasing public awareness of ocular trauma prevention, and formulating personalized prevention and control plans for different groups of people can effectively reduce the incidence and severity of ocular trauma.

## Data availability statement

The original contributions presented in the study are included in the article/supplementary material, further inquiries can be directed to the corresponding author.

## Ethics statement

The studies involving humans were approved by Medical Ethics Committee of Sichuan University. The studies were conducted in accordance with the local legislation and institutional requirements. Written informed consent for participation was not required from the participants or the participants' legal guardians/next of kin in accordance with the national legislation and institutional requirements.

## Author contributions

QC: Writing – original draft, Writing – review & editing. LL: Data curation, Investigation, Writing – original draft. YS: Data curation, Investigation, Writing – original draft. FL: Supervision, Writing – review & editing.
